# Prevalence of Healthcare-Associated Infections at the National Hospital During the COVID-19 Pandemic in Peru

**DOI:** 10.1017/ash.2021.154

**Published:** 2021-07-29

**Authors:** Jussara Huamani, Walter Prudencio

## Abstract

**Background:** Healthcare-associated infections are important because they constitute a public health problem due to the increase in morbidity and mortality that they produce in hospitalized patients, increased hospitalization costs due to prolonged stay, expensive antibiotic treatments and surgical reinterventions, not counting the social costs due to loss of wages and production, among others. **Methods:** We report the specific prevalence of healthcare-associated infections (HCAIs) in Edgardo Rebagliati Martin National Hospital, Perú, in 2020. We performed a descriptive cross-sectional study from July 27 to July 31, 2020. The medical records of hospitalized patients were reviewed according to the inclusion criteria. STATA software was used for descriptive statistical analyses. **Results:** In total, 1,217 hospitalized patients were included in the study. The prevalence of HCAI was 12.2% (149 patients). The prevalence of HCAI in areas where patients with the diagnosis of COVID-19 were hospitalized was higher (8.1%) than in common hospitalization areas (4.1%). Men represented 92% of the total number of patients with HCAIs. The most frequent infections were clinically defined pneumonia (30.9%) and bloodstream infections (20.1%). The most frequently isolated microorganism was *Pseudomonas aeruginosa*. **Conclusions:** The prevalence of HCAI was 12.2%. The most frequent HCAIs were pneumonia and bloodstream infection.

**Funding:** No

**Disclosures:** None

